# The effect of a post-anaesthesia high-care unit (PAHCU) admission on mobilization, length of stay and in-hospital mortality post-surgery in low energy neck of femur fracture patients

**DOI:** 10.1007/s00590-023-03799-1

**Published:** 2024-01-09

**Authors:** S. Essa, S. Venter, J. D. Jordaan

**Affiliations:** 1 Department of Anaesthesia and Critical Care, Faculty of Health Sciences, Stellenbosch University, Cape Town, South Africa; 2 Department of Orthopaedic Surgery, Faculty Health Sciences, Stellenbosch University, Cape Town, South Africa

**Keywords:** Fragility fracture of hip, Mortality, Mobilization, Discharge, PACHU

## Abstract

**Purpose/aim:**

With an ageing population and an increase in fragility fractures of the hip (FFH), the role of an anaesthetist is evolving to include more peri-operative care. A post-anaesthesia high-care unit (PAHCU) should enhance care in post-operative patients. To our knowledge, there are no studies that have investigated the effect of a PAHCU admission on post-operative outcomes after FFH. This study aimed to compare post-operative outcomes of FFH patients admitted to PAHCU versus a standard post-operative orthopaedic ward (POOW).

**Methodology:**

A retrospective cohort study was conducted on adult patients with FFH who underwent surgery between January 2019 and December 2020 at our institution. Data were sourced from electronic medical records. SPSS version 28 was used to analyse data.

**Results:**

A total of 231 patients were included. The PAHCU group (*n* = 35) displayed a higher burden of chronic illness and higher peri-operative risk scores as compared to the POOW group (*n* = 196). Median time to mobilize (TTM) in PAHCU was 84 h vs. 45 h in POOW group (*p* = 0.013). Median length of stay (LOS) in PAHCU was 133 h vs. 94 h in POOW (*p* = 0.001). The in-hospital mortality was 2.9% (*n* = 1) for PAHCU and 3.6% (*n* = 7) for POOW (*p* = 1). The 30-day mortality was 11.8% (*n* = 4) for PAHCU and 10.1% (*n* = 19) in POOW.

**Conclusion:**

PAHCU admission resulted in delayed time to surgery and TTM, together with prolonged LOS, compared to those managed in POOW. However, these mortality rates remained comparable in both groups. This study contributes valuable insights into post-operative care of FFH patients in a resource-poor setting.

## Introduction

Fragility fractures of the hip (FFH) pose a significant economic burden on global health. With the worldwide rise in the elderly demographic, the total number of hip fractures is projected to nearly double over the next 20–30 years [1].

A FFH introduces an acute physiological stress, challenging the resilience of patients already compromised by advanced age and comorbid illness. These frail patient phenotypes are at high risk of peri-operative complications and fatal adverse events [2]. Despite optimal surgical intervention, only 41–67% of FFH patients regain their pre-fracture ambulatory ability within a year [3]. A Systematic review showed in-hospital mortality rate between 3 and 7%, 30-day mortality of 6.1–8.7% and one-year mortality of 2.4–34% [4]. Factors such as advanced age, male sex, comorbidities, place of residence, cognitive impairment, time to surgery, and low mean arterial pressure have been identified as significant predictors of post-operative mortality in FFH patients [5, 6].

Recent studies from South Africa (SA) reported 3.3% in-hospital mortality rate, 5.6% at 30 days and 26.7% at 1 year [7]. The incidence rate for FFH in SA is 68.6 patients per 100,000 population, occurring at a younger average age (60 years) than the international averages. The high prevalence of HIV, along with antiretroviral treatment, is thought to affect bone metabolism and increase risk of secondary osteoporosis [8, 9].

Considering the socioeconomic and health burden resulting from FFH, health authorities and clinicians worldwide have produced best practice guidelines [10]. These include the development of enhanced recovery after surgery (ERAS) protocols and multidisciplinary orthogeriatric units to optimize peri-operative care and facilitate early mobilization [6, 11, 12].

Anaesthetists, as peri-operative physicians, are a crucial part of the multidisciplinary team. Anaesthetic guidelines for FFH management have shown conflicting recommendations [13]. The Association of Anaesthetists and the Fragility Fracture Network address some of these inconsistencies in their latest best practice guidelines [6]. An unresolved controversy is the level of post-operative care required for these patients. Intensive care unit (ICU) care is often unfeasible, particularly in resource-constrained environments such as in SA. A post-anaesthesia high-care unit (PAHCU) provides a higher level of post-operative care than a standard post-operative orthopaedic ward (POOW) but is far less resource-intensive than an ICU.

The POOW is a 40-bed ward with only one sister, two staff nurses, and monitoring occurs 6 hourly. PAHCU is a high-care unit that admits patients for less than 24 h [14]. It is separate from ICU but attached to the theatre complex. Patient management is overseen by the anaesthetic department. It provides care for patients requiring less ventilatory support, optimizing pain management in a less stimulating environment [14, 15]. The impact of PAHCU after FFH surgery has been minimally investigated worldwide despite the perceived benefits [16]. A Swedish study demonstrated decreased length of stay (LOS) and in-hospital mortality of post-surgical patients following the implementation of a clinical pathway in PAHCU [16]. No similar studies have been conducted in the African context.

The main aim of this study was to determine the effect of a PAHCU admission on LOS, time to mobilize (TTM) and mortality outcomes post-surgery in FFH patients, as compared to care in a POOW.

## Methodology

### Study design

A retrospective cohort study was undertaken at a single tertiary hospital in SA. All patients who presented with a FFH and received arthroplasty surgery were included for the two-year period, January 2019 to December 2020. The a priori sample size was calculated based on the primary objective. We assumed an allocation ratio of 0.2 between the two groups (49 patients from the PAHCU and 245 patients from the POOW). At 80% power and alpha level of 0.05, approximately 300 patients will allow an effect size (Cohen’s D) of 0.44 to be detected based on an independent samples *t* test.

Patients younger than 45 years, high-energy trauma, revisions, multiple fractures, and pathological fractures were excluded. We included 231 patients for analysis. The primary objective analysis showed a *R*^2^ = 0.05 based on a Mann–Whitney test, with a *p* value = 0.001, thus providing adequate power at *n* = 231.

An anaesthetist assessed all patients prior to surgery and patients were placed either in PAHCU (for 24 h) or into a POOW. Selection criteria for PAHCU were determined by availability of a bed and the anaesthetists clinical judgement of peri-operative risk. The following information was collected and compared: age, gender, comorbidities, American Society of Anaesthesiologists (ASA) classification, type of surgery/anaesthetic, Revised Cardiac Risk Index (RCRI), time between admission and surgery, troponin blood results pre- and post-surgery, TTM by physiotherapy, pre-ambulatory status, LOS (readiness for discharge), and in-hospital and 30-day mortality.

Physiotherapy was initiated in a haemodynamically stable patient, after X-ray confirmation of a stable implant fit for partial or total weight bearing. No physiotherapy services were available in PAHCU. Readiness for discharge was determined by physiotherapy in collaboration with orthopaedics when patients met pre-determined mobility goals based on their pre-operative ambulatory status.

Data were collected from the orthopaedic database and the electronic hospital patient records. Waiver of informed consent was approved by the institutional ethics committee. All data were anonymized and entered into MS Excel.

### Statistical analysis

SPSS version 28 was used to analyse data. Demographic and clinical features of both groups were described using frequency tables and summary statistics and compared using chi square tests in the case of categorical variables, whilst *t* tests or Mann–Whitney tests were used to compare measured variables between the two groups. A *p* value < 0.05 was used to indicate statistical significance.

The Mann–Whitney test was used to compare the distribution of time to discharge and TTM between the two groups since these variables were not normally distributed (nonparametric).

In order to test the secondary hypothesis, the Fisher’s 2-sided exact test was used to compare mortality counts between the two groups. Ethical approval was obtained from Stellenbosch University Committee for Human Research (HREC). The study was conducted in accordance with the declaration of Helsinki and the Department of Health Guidelines for Good Clinical Practice. Hospital consent was obtained.

## Results

The final cohort included 231 participants, mostly females 63% (*n* = 146), with an average age of 73 years (range 47–99). Patients admitted to PAHCU were slightly older, 79 years old (SD =  ± 11.5) compared to the average POOW patient age of 72 (SD =  ± 12). Post-operative destination was PAHCU for 15% (*n* = 35) of patients and 85% (*n* = 196) of patients went to POOW.

**Fig. 1:**
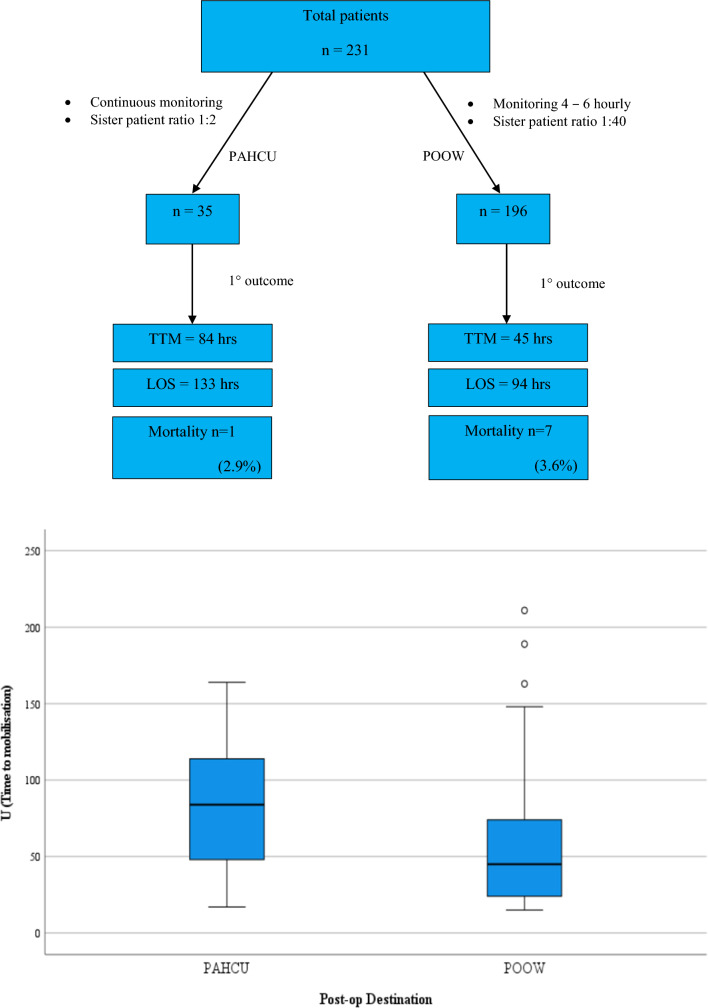
Depicting difference in time to mobilization

**Fig. 2:**
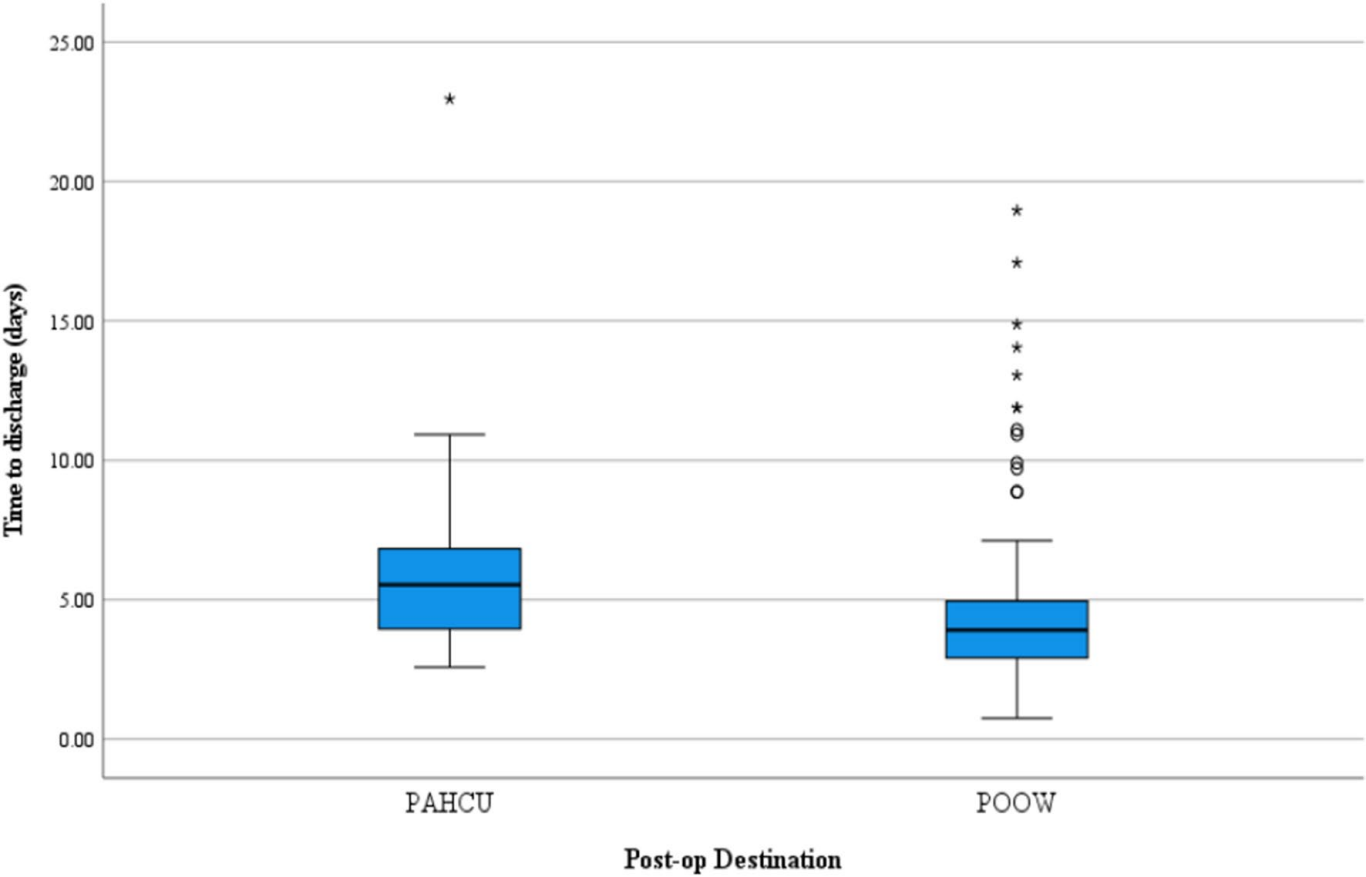
Depicting difference in time to discharge

The median TTM in PAHCU vs. POOW was 84 and 45 h, respectively, (*p* = 0.013). The median time to discharge in PAHCU vs. POOW was 133 and 94 h, respectively, (*p* = 0.001). The time from admission to surgery in PAHCU vs. POOW was 97 and 61 h, respectively, (*p* = 0.008) (Figs. 1, 2).

The total in-hospital mortality was 3.5% (*n* = 8), 2.9% (*n* = 1) for PAHCU and 3.6% (*n* = 7) for POOW (*p* = 1). The 30-day mortality was 10.3% (*n* = 23), 11.8% (*n* = 4) for PAHCU and 10.1% (*n* = 19) in POOW (*p* = 1).

The surgical management was mostly a bipolar hemi-arthroplasty in 63% of group (*n* = 114), PAHCU 88% (*n* = 30) and POOW 58% (*n* = 114). Surgery was performed mainly via the direct anterior approach (94%).

The majority of FFH received regional anaesthesia in the form of a spinal or epidural, 39% (*n* = 13) of PAHCU sample had a spinal vs. 89% (*n* = 167) of POOW patients. PAHCU group received an epidural in 39% (*n* = 13) of cases vs. 2% (*n* = 4) of POOW group.

FFH patients were 37% (*n* = 83) ASA2 and 53% (*n* = 118) ASA3. No ASA1 patients were admitted to PAHCU. Eighty-five percentage (*n* = 28) of PAHCU sample were ASA3 as opposed to 48% (*n* = 90) in POOW (*p* = 0.002).

**Fig. 3:**
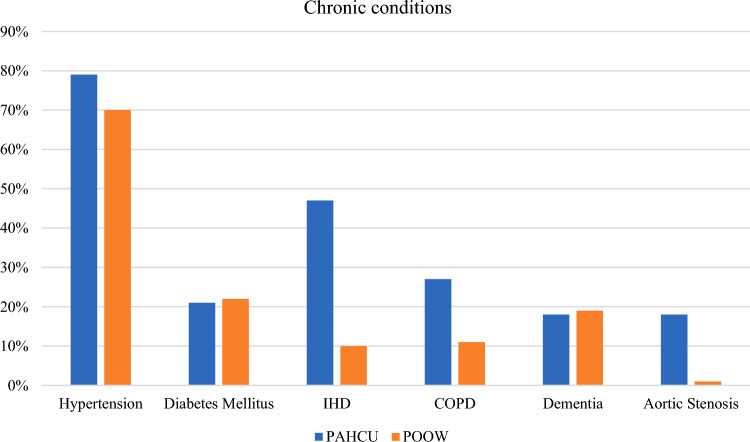
Common comorbidities and differences between PAHCU and ward. *IHD* ischaemic heart disease, *COPD* chronic obstructive pulmonary disease

The most common chronic conditions were hypertension, 69% (*n* = 159), diabetes mellitus (DM), 21% (*n* = 49), and dementia, 19% (*n* = 42). The figures were comparable amongst the two groups. Nevertheless, 47% of PAHCU sample had IHD compared to 10% (*n* = 34) POOW patients. Twenty-seven percent (*n* = 9) of PAHCU patients had COPD compared to 11% (*n* = 21) of those in POOW. Eighteen percent (*n* = 6) of PAHCU sample had aortic stenosis (AS) vs. 1% (*n* = 2) of POOW group (Fig. 3).

Majority, 67% (*n* = 148) of patients had a RCRI0 score and they mainly got admitted to POOW. A significantly higher proportion of patients with RCRI between 1 and 3 were admitted to PAHCU than POOW (*p* < 0.001). There were no patients with a RCRI score greater than 3 that went to PAHCU.

### Troponins

Pre-operative troponins testing was not routinely performed and only 44 patients were included. As per the *VISION* trial, an absolute increase between pre- and post-operative levels > 5 was used as significant if troponins were between 20 and 65 [17] In PAHCU 27% (*n* = 3) vs. 42% (*n* = 14) of POOW group had increased troponin levels, however, this was not statistically significant (*p* = 0.486) (Tables 1 and 2).

**Table 1:** Depicting demographics and main outcomes

	PAHCU	POOW	*p* value
Number of patients (*n* = 231)	35	196	
Age (mean)	79 (SD = ± 11.5)	72 (SD = ± 12)	
Gender			
Male	9	76	0.14
Female	26	120	
Time: (median in h)			
Mobilization (*n* = 148)^a^	84	45	0.013
To discharge (*n* = 208)^a^	133	94	0.001
From admission to surgery (*n* = 217)^a^	97	61	0.008
In-hospital mortality (*n* = 231)	1 (2.9%)	7 (3.6%)	1
30-day mortality (*n* = 223)	4 (11.8%)	19 (10.1%)	1

*p* < 0.05 is statistically significant

^a^*n* differed in categories since not all records were found

**Table 2:** Depicting differences between PAHCU and ward

	PAHCU	POOW	*p* value
Type of surgery^a^	(*n* = 34)	(*n* = 196)	< 0.001
Bipolar	30	114	
THR	4	82	
Surgical approach^a^	(*n* = 34)	(*n* = 195)	0.247
Anterior	33	182	
Posterior	1	13	
Anaesthetic type^a^	(*n* = 33)	(*n* = 188)	< 0.001
Spinal	13 (39%)	167 (89%)	
Epidurals	13 (39%)	4 (2%)	
CSE	1 (3%)	0 (0%)	
General	6(18%)	17 (9%)	
Co-morbidities^a^			
Hypertension	26 (74%)	133 (68%)	0.515
DM	7 (20%)	42 (21%)	0.827
IHD	15 (47%)	19 (10%)	< 0.001
Dementia	6 (18%)	36 (19%)	0.538
COPD	9 (27%)	21 (11%)	0,021
Aortic stenosis	6 (18%)	2 (1%)	< 0.001
ASA^a^	(*n* = 33)	(*n* = 188)	< 0.01
1	0	6 (3%)	
2	2 (6%)	81 (43%)	
3	28 (85%)	90 (48%)	
4	3 (9%)	10 (5%)	
5	0	1 (0.5%)	
RCRI^a^	(*n* = 32)	(*n* = 189)	< 0.001
0	10 (31%)	137 (73%)	
1	15 (47%)	36 (19%)	
2	6 (19%)	12 (6.4%)	
3	1 (3%)	1 (0.5%)	
4	0	2 (1%)	
5	0	1 (0.5%)	
6	0	0	
Troponins (*n* = 44)			0.486
Increase by 5 if 20–65	3 (27%)	14 (42%)	

*p* < 0.05 is statistically significant

^a^*n* differed in categories since not all records found

## Discussion

There is a large variation in peri-operative management of FFH patients worldwide. To our knowledge, this is the first study investigating the perceived differences in outcome between FFH patients admitted to a PAHCU vs. a POOW. In SA there are no geriatric-led multidisciplinary FFH teams. This results in increased peri-operative care by the Anaesthetist and Orthopaedic surgeon to facilitate safe and reproducible outcomes in our resource-constrained environment.

Our analysis of peri-operative risk stratification and comorbid illness indicators revealed that the PAHCU sub-group bears a higher burden of illness and risk. This is as anticipated, since PAHCU bed space is typically allocated to patients with greater perceived peri-operative risk. Notably, 89% of PAHCU patients were classified as ASA3 or above, compared to 54% of POOW patients. There were also statistically significant RCRI score differences between post-operative destinations (*p* < 0.003), with PAHCU group exhibiting higher scores. The RCRI aids in predicting major cardiac events in adults undergoing significant non-cardiac surgery.

Hypertension, DM, and Dementia had a similar prevalence in both groups, however, AS, IHD, and COPD were more prevalent in the PAHCU sample. It is clear from these findings that the PAHCU sub-group could, by conventional measures, be considered at higher peri-operative risk than the POOW sub-group.

Our study measured three distinct outcomes: time to mobilization (TTM), length of stay (LOS) and mortality (in-hospital and 30-day).

Median TTM was significantly prolonged in the PAHCU patient sample. This is concerning, since early post-operative ambulation (day 0–1) has been linked to reduced incidence of delirium, pneumonia, improved functional outcomes, and lower mortality [10].

Hospital LOS was found to be significantly different between the two groups. PAHCU patients had a median LOS of 133 h, compared to 94 h for POOW group (*p* = 0.001). These values are markedly lower than the mean of 11 days reported in other studies [18]. The reasons for this discrepancy are unclear. Many developed countries enforce strict discharge criteria and frequently conduct in-house rehabilitation [19]. Nevertheless, in overburdened healthcare settings such as our South African context, earlier discharge may be the most practical option. Another contributing factor was that the surgical approach for FFH in this study was via the direct anterior approach with associations of less pain and early functional recovery. It is important to note that discharge-readiness was used as a surrogate for LOS in our study to avoid inclusion of any logistic reasons for delayed discharges which could confound results.

These discrepancies between our patient groups can likely be attributed to several overlapping factors. The PAHCU sub-group frequently has invasive lines, epidurals, or urinary catheters that restrict movement. Another potential reason for the delay at our facility is the absence of physiotherapy services in PAHCU. This highlights a critical issue that warrants further investigation and possible adjustments to enhance patient outcomes in our institution, especially since respiratory failure is the most common cause of in-hospital mortality in FFH patients [20].

It is noteworthy that there was no significant difference in in-hospital mortality between the two groups. (2.9% PAHCU vs 3.6% POOW) This figure aligns with international standards [4, 20]. Similarly, there was no difference in 30-day mortality between these two groups (*p* = 1), with rates of 11% for PAHCU versus 10% for POOW. Whilst these figures are higher than the global mortality rates reported at 6–8.7%, they align with the statistics reported in SA [4, 8].

Patients admitted to a PAHCU post FFH surgery, with a higher ASA and RCRI scores, are expected to have an increased mortality rate, compared to lower-risk patients managed in a standard POOW. The similar mortality rates between the two groups could not be explained by our study design. A possible hypothesis could be related to the peri-operative care offered by our PAHCU team which includes an in-house anaesthetist in the role of a peri-operative physician. Alternatively, the discrepancy might stem from the inadequacy of current methods to risk-stratify patients with FFH. Numerous other possibilities exist, underscoring the need for further studies with larger sample sizes to provide more definitive answers.

The statistically significant difference in median time from admission to surgery between PAHCU and POOW is noted (*p* = 0.008).

Time to surgery is critical and has been shown to impact early mobilization, morbidity, and mortality of FFH patients. A meta-analysis revealed that those who were operated within 48 h of admission experienced significantly lower mortality [10]. The *Hip Attack* trial showed that surgery within 6 h of diagnosis, reduces delirium, urinary tract infections, TTM, and hospital LOS [21]. The exact reasons for the prolonged time to surgery for both groups could not be ascertained in this study. A plausible explanation could be that we function in a resource-constrained environment in which the FFH patients compete for theatre space with other surgical disciplines. The extended delay in surgery in the PAHCU cohort could be due to unavailable PAHCU beds.

This creates a dilemma of whether to postpone surgery until a bed is available, assuming improved care or whether to proceed and send patient to the POOW.

Regardless of the reasons for the delay, this study demonstrated that admission to PAHCU did not negatively impact either in-hospital or 30-day mortality. This finding is noteworthy, particularly as the PAHCU cohort was presumed to be at a higher peri-operative risk and yet demonstrated similar mortality outcomes in this study. It should be noted, however, that the time to surgery in both groups exceeded the recommended 48-h limit. This could potentially affect or confound our results, presenting an opportunity for further research to bring clarity to these questions.

The high prevalence of dementia, 19% (*n* = 42) in this population merits mention. It is unsurprising that dementia is common in this elderly group of patients, with international figures reaching up to 23% [22]. The association between dementia, post-operative delirium and increased 30-day mortality rates has long been recognized [22]. These findings might prompt orthopaedics to utilize current risk stratification tools for dementia scoring, which are underutilized in our institution. In our cohort, we could not correlate peri-operative dementia with increased mortality rates.

This study addresses the scarcity in African Literature on the management of FFH patients and provides a unique perspective from a resource-limited setting.

Potential limitations include the retrospective design, with some records missing, the single centre cohort and the relatively small sample size. The Covid-19 pandemic may also have influenced the results.

## Conclusion

FFH patients that are selected via risk stratification by anaesthetists for PAHCU will have a delay in time to surgery, TTM and a prolonged LOS, compared to FFH patients treated in a POOW. However, both the in-hospital and 30-day mortality rates remain comparable in both groups.

A multidisciplinary peri-operative team, comprising of the surgeon, anaesthetist, and physiotherapist can anticipate good outcomes in our resource-limited setting comparable to centres which have orthogeriatric services available.

This study contributes valuable insights into the post-operative care of FFH patients in a resource-limited setting, filling a gap in the existing literature, and offering a foundation for future research and decision-making regarding resource allocation and patient care optimization.

## References

[1] Sing CW, Lin TC, Bartholomew S (2023). Global epidemiology of hip fractures: secular trends in incidence rate, post-fracture treatment, and all-cause mortality. J Bone Miner Res.

[2] Baroni M, Serra R, Boccardi V (2019). The orthogeriatric comanagement improves clinical outcomes of hip fracture in older adults. Osteoporos Int.

[3] Uhm KE, Yoo JS, Chung SH (2017). Effects of exercise intervention in breast cancer patients: is mobile health (mHealth) with pedometer more effective than conventional program using brochure?. Breast Cancer Res Treat.

[4] Downey C, Kelly M, Quinlan JF (2019). Changing trends in the mortality rate at 1-year post hip fracture - a systematic review. World J Orthop.

[5] Morri M, Ambrosi E, Chiari P (2019). One-year mortality after hip fracture surgery and prognostic factors: a prospective cohort study. Sci Rep.

[6] Shelton C, White S (2020). Anaesthesia for hip fracture repair. BJA Educ.

[7] Jordaan JD, Sa HDO, Sa FCO (2022). Mortality rates in femoral neck fractures treated with arthroplasty in South Africa. Geriatr Orthop Surg Rehabil.

[8] Dela SS, Paruk F, Brown SL (2020). Ethnic and gender-specific incidence rates for hip fractures in South Africa: a multi-centre study. Bone.

[9] Swenning T, Leighton J, Nentwig M, Dart B (2020). Hip fracture care and national systems. OTA Int Open Access J Orthop Trauma.

[10] Ferris H, Brent L, Coughlan T (2020). Early mobilisation reduces the risk of in-hospital mortality following hip fracture. Eur Geriatr Med.

[11] Liu S, Li C, Zhang P (2021). Enhanced recovery after surgery for hip fractures: a systematic review and meta-analysis. Perioper Med.

[12] Gupta A (2014). The effectiveness of geriatrician-led comprehensive hip fracture collaborative care in a new acute hip unit based in a general hospital setting in the UK. J R Coll Physicians Edinb.

[13] Kearns RJ, Moss L, Kinsella J (2013). A comparison of clinical practice guidelines for proximal femoral fracture. Anaesthesia.

[14] Harmse L, Ahmed N, Cilliers C (2023). The utilisation of the post-anaesthesia high-care unit at Tygerberg Hospital: a retrospective audit. S Afr J Anaesth Analg.

[15] Davidson M, Litchfield K (2018). Patient recovery and the post-anaesthesia care unit (PACU). Anaesth Intensive Care Med.

[16] Åhman R, Siverhall PF, Snygg J (2018). Determinants of mortality after hip fracture surgery in Sweden: a registry-based retrospective cohort study. Sci Rep.

[17] Devereaux M (2017). Association of postoperative high sensitivity troponin levels with myocardial injury and 30-day mortality among patients undergoing noncardiac surgery. JAMA.

[18] Ntuli M, Filmalter C, White Z, Heyns T (2020). Length of stay and contributing factors in elderly patients who have undergone hip fracture surgery in a tertiary hospital in South Africa. Int J Orthop Trauma Nurs.

[19] Li G, Yu F, Liu S (2023). Patient characteristics and procedural variables are associated with length of stay and hospital cost among unilateral primary total hip arthroplasty patients: a single-center retrospective cohort study. BMC Musculoskelet Disord.

[20] Groff H, Kheir MM, George J (2020). Causes of in-hospital mortality after hip fractures in the elderly. HIP Int.

[21] Borges FK, Bhandari M, Guerra-Farfan E (2020). Accelerated surgery versus standard care in hip fracture (HIP ATTACK): an international, randomised, controlled trial. Lancet.

[22] Ioannidis I, Mohammad Ismail A, Forssten MP (2022). The mortality burden in patients with hip fractures and dementia. Eur J Trauma Emerg Surg.

